# Endocarditis after Transcatheter Aortic Valve Replacement

**DOI:** 10.3390/jcm12227042

**Published:** 2023-11-11

**Authors:** Lorenzo Braghieri, Simrat Kaur, Christopher K. Black, Paul C. Cremer, Shinya Unai, Samir R. Kapadia, Amgad Mentias

**Affiliations:** 1 Department of Internal Medicine, Cleveland Clinic Foundation, Cleveland, OH 44195, USA; braghil@ccf.org (L.B.); black4@ccf.org (C.K.B.); 2 Heart, Vascular and Thoracic Institute, Cleveland Clinic Foundation, Cleveland, OH 44195, USA; kaurs4@ccf.org (S.K.); paul.cremer@gmail.com (P.C.C.); unais@ccf.org (S.U.); kapadis@ccf.org (S.R.K.)

**Keywords:** transcatheter aortic valve replacement, infective endocarditis, TAVR, SAVR, aortic valve replacement

## Abstract

Transcatheter aortic valve replacement (TAVR) use is gaining momentum as the mainstay for the treatment of aortic stenosis compared to surgical aortic valve replacement (SAVR). Unfortunately, TAVR-related infective endocarditis (TAVR-IE) is expected to be detected more and more as a result of the ever-expanding indications in younger patients. Given the overall poor prognosis of TAVR-IE, it is imperative that clinicians familiarize themselves with common presentations, major risk factors, diagnostic pitfalls, therapeutic approaches, and the prevention of TAVR-IE. Herein, we review all of the above in detail with the most updated available literature.

## 1. Introduction

For individuals above 75 years old, aortic sclerosis is common, with a prevalence of about 40%, and ~2%/year progress to hemodynamically significant aortic stenosis (AS) [1]. Transcatheter aortic valve replacement (TAVR) is an established therapy across risk profiles for severe native AS and is also indicated in bioprosthetic aortic valve failure [2,3,4,5,6]. In addition, given promising short-term results, there is an emerging role for TAVR in high-surgical-risk candidates with pure severe native aortic valve regurgitation [7,8].

As long-term durability data become available and operators accumulate experience with valve-in-valve TAVR (ViV-TAVR), TAVR use may increase in middle-aged individuals. With this potential shift, there is a need to better understand valve-related risks over the next few decades. A central concern is prosthetic valve endocarditis, which is rare but carries significant morbidity and mortality [9,10]. Our narrative review aims to provide a scoping overview of the most common risk factors and causative organisms, major diagnostic challenges, evidence-based therapeutic approaches and prophylactic strategies of TAVR-related infective endocarditis (TAVR-IE). This review also aspires to foster collaborative, multi-center research in the field of TAVR-IE with the goal of improving outcomes.

## 2. Epidemiology

The overall incidence rate of TAVR-IE is low, ranging from 0.9 to 1.7 events per 100 person-years, but still accounts for a high proportion of all surgical explants of TAVR valves [9,10,11,12,13]. Differences in study designs and included patient populations account for the relatively wide variation in reported TAVR-IE incidence. For instance, non-uniform TAVR-IE diagnosis criteria or distinct diagnostic modalities were employed across several databases. In addition, the TAVR patient population has continuously evolved with expanding indications over the last decade, possibly contributing to the different reported incidence rates of TAVR-IE. In particular, a significant shift was represented by the inclusion of younger low-surgical-risk patients [9]. Notably, a recent study demonstrated that the incidence of TAVR-IE in a small cohort of low-surgical-risk patients was equivalent to intermediate- and high-risk patients [14]. However, the incidence of TAVR-IE in a large US nationwide cohort has decreased from 2012 to 2017, which is likely due to the expansion to lower-risk patients and advancements in prosthesis design [10]. Ongoing studies are needed to characterize the impact of this growing TAVR patient subpopulation on the incidence of TAVR-IE, which may continue to be reshaped in the coming years [15].

The incidence of TAVR-IE across time has been well characterized (Table 1).

Although higher in the period immediately after TAVR, TAVR-IE still occurs one year after the procedure. Based on randomized data on low-risk TAVR patients, the incidence of very early (i.e., <30 days post-TAVR), early (i.e., between 31 and 365 days post-TAVR), and late (i.e., > 1-year post-TAVR) TAVR-IE is 0%, 1.5% and 2.8%, respectively [14]. Of note, both PARTNER 3 and the Evolut trial showed a similar incidence of early TAVR-IE, but a lower incidence of early TAVR-IE (0.2% for both) [5,6]. Del Val et al. compared the “historical” TAVR (i.e., prior to 2014) to the more “contemporary” TAVR (i.e., after 2014), finding that TAVR-IE in the first 60 days was lower in the contemporary group (2.3 vs. 4.9 per 1000 patient-years) [16]. Improved operators’ skills, standardized post-operative care as well as the inclusion of lower-risk and younger patients might be plausible causes [16]. Of note, the incidence of TAVR-IE after 60 days post-TAVR did not vary between the “contemporary” and “historical” groups [16]. A similar trend was also reported in an updated low-surgical-risk cohort with a lower incidence of TAVR-IE in the first 30 days [14]. Overall, the incidence of TAVR-IE across all groups appears to be higher in the first year when compared to later after the first year [10,11,14,16]. In particular, the large Swiss TAVR observational cohort study showed that the incidence of TAVR-IE was highest in the first 100 days after the procedure [17]. The risk of hospital-acquired infections from multiple sources (e.g., central lines or urinary catheters), the vulnerable post-operative period, as well as a slow host endothelium growth, are likely to contribute to this reported early risk.

Although TAVR is less invasive than SAVR, large multicenter studies have suggested that they have similar overall incidence of IE [9,18]. Interestingly, the rate of early endocarditis (within 30 days) is similar in both TAVR and SAVR [9,12]. Furthermore, the incidence (1.7% vs. 1.9% per person-year) and median time (91 vs. 92 days) to endocarditis were similar between TAVR and SAVR in an extensive propensity-matched analysis [12]. It is important to note that this study included older TAVR prostheses and did not include TAVR patients beyond 2014. In the updated pooled analysis of PARTNER 1 and PARTNER 2 trials, the incidence and temporal risk of IE were again found to be similar between TAVR and SAVR [9].

## 3. Risk Factors

Although many risk factors for TAVR-IE have been described, the strength of associations has been modest. Therefore, reported risk factors have been clinically useful for broad risk stratification rather than modifiable patient-specific factors. In general, risk factors are divided into two categories related to patient characteristics and procedure-related factors.

### 3.1. Patient-Related Risk Factors

Male sex is associated with a higher risk of TAVR-IE [10,11,13,17]. This association is similar to endocarditis in the general population including native valve endocarditis, where men are affected more than women [19]. A broad spectrum of patient comorbidities has been associated with an increased risk of TAVR-IE. The following have been observed to increase the risk of TAVR-IE with an increased relative risk ranging from 71% to 39%: renal insufficiency, pulmonary disease, prior endocarditis, pacemaker placement, diabetes, preexisting atrial fibrillation, intravenous drug use, heart failure, and liver disease [9,10,11,12].

### 3.2. Procedure-Related Risk Factors

Importantly, several components related to the TAVR procedure itself can significantly contribute to TAVR-IE. For instance, skipping balloon pre-dilatation or optimizing prosthesis implant via balloon post-dilation (BPD) is associated with a 49% and 83% increased risk of TAVR-IE, respectively [9,17]. Pre-dilatation is performed to increase the orifice area, allowing for easier implantation and uniform expansion of the TAVR prosthesis. While particularly helpful in challenging valve anatomy (e.g., bicuspid or severely calcified valves), direct valve implantation is more frequently feasible with successive iterations of TAVR devices. However, avoiding pre-dilatation may lead to mild paravalvular leaks with high-velocity flow causing endothelial damage, especially with TAVR prostheses with lower radial force such as self-expanding valves (SEV). Paravalvular leakage is a risk factor for TAVR-IE [20]. Despite advances in TAVR prostheses design, BPD is still frequently performed with the goal of reducing paravalvular leak severity and optimizing frame expansion. BPD may increase the risk of IE by microscopically damaging valve leaflets, thereby promoting platelet aggregation and bacterial seeding. In addition, smaller studies reported differences in the incidence of TAVR-IE between different valve platforms, but large studies did not show any significant difference between balloon-expandable valves (BEVs) and SEVs [11]. 

Furthermore, research has focused on the impact of transfemoral access since *Enterococcus*, which is commonly found in the groin, has been frequently found as a causative agent of TAVR-IE [9,11,16]. The prevalence of Enterococcal TAVR-IE has been attributed to the close proximity to the genitourinary system of transfemoral access [21,22]. However, transfemoral access has not been shown to increase the risk of TAVR-IE compared to the alternative transthoracic approach [9,23]. In a nationwide study, Bjursten et al. found that the transapical approach increased the risk of TAVR-IE [24]. 

Finally, the risk of TAVR-IE may be influenced by the native aortic valve anatomy and early post-TAVR results. For example, Bjursten et al. observed a 2% increased relative risk of early TAVR-IE in severely calcified valves for every 1 mmHg increase in pre-procedural mean gradients, possibly from the need to fracture the heavily calcified valve leaflets resulting in foci of endothelial damage [24]. However, this was not observed to contribute to late TAVR-IE, suggesting that appropriate antibiotic prophylaxis minimizes the risk of TAVR-IE as the endothelium heals [11,24]. For similar reasons, ViV-TAVR was also associated with a 62% increased risk of TAVR-IE [10]. Given the insidious presentation of prosthetic valves IE, it is worth mentioning that the high incidence of TAVR-IE after ViV-TAVR may also be attributed to unrecognized IE as the original cause of valve dysfunction. Conversely, post-TAVR moderate-severe aortic regurgitation is associated with a 2-fold increased risk of TAVR-IE [11], which is likely the consequence of high sheer stress from turbulent blood flow favoring the deposition of fibrin and the creation of a nidus for infection [25].

## 4. Microbiology

Soft tissue infections and intravascular access have been the most frequently identified sources of TAVR-IE [11]. However, the source of bacteremia may not be identified in up to 50% of TAVR-IE cases [26]. Culture-negative TAVR-IE is rare compared to native valve IE and SAVR-related IE (SAVR-IE). On the other hand, *Enterococcus*, *Staphylococcus*, and *Streptococcus* were identified as the causal organisms in over two-thirds of TAVR-IE similar to native valve IE. These three organisms have accounted for most TAVR-IE cases in prior studies [9,10,11,13,27]. The multi-center Swiss TAVI Registry reported no significant differences in causative microorganisms among different TAVR-IE timelines [17]. However, *Enterococcus* faecalis was the predominant bacteria in very early and early TAVR-IE, while *Staphylococcus* aureus and viridans-group *Streptococci* were the most common bacteria in late TAVR-IE. Individual risk factors for the most and least common pathogens are summarized in Table 2.

Unlike SAVR-IE, some studies report *Enterococcus* as the most frequent causal organism in TAVR-IE [11,16,17]. Advanced age and multiple comorbidities, which were typical features of the historical TAVR cohort, may favor groin colonization by *Enterococcus* due to frequent healthcare use and higher antibiotic exposure. *E. faecalis* was the most commonly reported species in an early TAVR cohort, accounting for 65.8% of these cases [26]. 

*Staphylococcus* and *Streptococcus* are the other most common causative organisms in TAVR-IE after *Enterococcus*. Among *Staphylococcus* genus, *S. aureus* accounts for 60% of the observed subspecies [26]. Compared to other causative organisms, *S. aureus* has demonstrated a higher virulence in TAVR-IE patients with an in-hospital mortality of 47.8% and a 2-year mortality of 71.5% [12,18]. Compared to other organisms, *S. aureus* is associated with soft tissue infection and vascular access, which account for 8.5% and 9.2% of *S. aureus* TAVR-IE, respectively [28]. *Streptococcus* is also a common cause of TAVR-IE but is less prevalent in TAVR-IE compared to SAVR-IE [11,12,18]. Over the last two decades, healthcare-associated TAVR-IE has become increasingly common and has been present in about 50% of TAVR-IE [11]. With this high prevalence, an increase in drug-resistant bacteria such as methicillin-resistant *S. aureus* (MRSA) has been observed. For instance, in a 2018 study by Kolte et al., one-third of all *Staphylococcus* cases were secondary to MRSA [12].

Less common causes of TAVR-IE include Gram-negative (GN) organisms and fungal infections. GN-related TAVR-IE incidence is up to 5% with a median time to TAVR-IE of 1.1 months [26], hinting at nosocomial infections as potential origin. In fact, 17% of patients develop nosocomial infections following TAVR with GN organisms isolated in 60% of blood cultures [29]. The incidence of fungal TAVR-IE ranges from 0.8–3% and is associated with a substantially increased risk of mortality [17,22].

## 5. Presentations and Diagnosis

**Figure 1:**
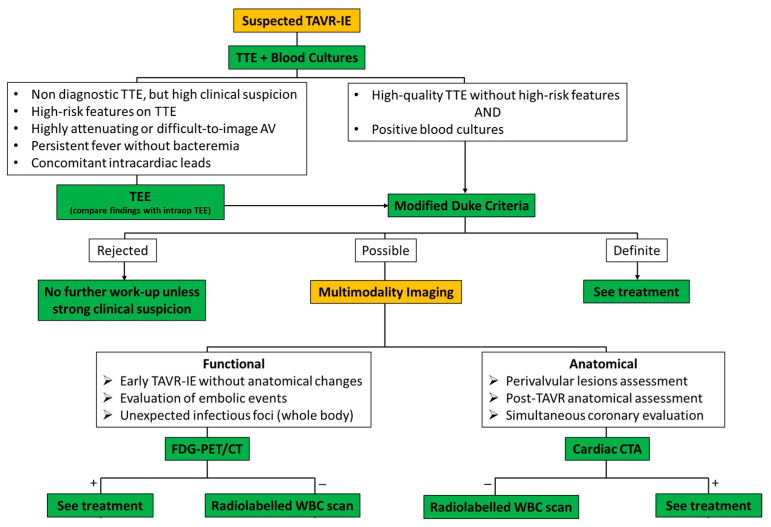
Proposed diagnostic algorithm of TAVR-related IE (TAVR-IE). TTE, transthoracic echocardiography; AV, aortic valve; TEE, transesophageal echocardiography; FDG-PET/CT, 18F-fluorodeoxyglucose positron emission tomography; CTA, computed tomography angiography; +/−, positive or negative test results.

In general, the pathoanatomical presentation of IE is diverse and largely dependent on the involved organism. Valve involvement may range from relatively benign minor bacterial colonization to abscess or fistula formation up to the destruction of the valve apparatus. The presentation of TAVR-IE and native valve IE share common presenting symptoms, but there are key differences that should be considered in the assessment of suspected TAVR-IE. A diagnostic algorithm is shown in Figure 1.

The most common presenting symptom of TAVR-IE is fever, but patients also may present with new-onset heart failure and systemic embolism [11,16]. Especially when considering the high comorbidity burden of the “historical” TAVR population, the classic symptoms of TAVR-IE may be initially overlooked. To make diagnosis more challenging, atypical presentations occur more frequently in TAVR-IE compared to native valve IE. For instance, although fever is still the most common presenting symptom [11,16], the contemporary TAVR cohort may present without fever in about 30% of cases [16]. The lack of cardinal native valve IE signs and symptoms may lead to a delayed diagnosis with catastrophic presentations and possibly contribute to the poor outcomes of TAVR-IE [10].

From a diagnostic imaging standpoint, numerous echocardiographic findings have been reported, including vegetations, paravalvular lesions, new regurgitation, leaflet thickening, and increased mean transvalvular gradients [30]. Of note, isolated prosthesis infection has been reported only in 48% of cases, with concomitant mitral valve, tricuspid valve, or pacemaker lead infection in 20%, 11%, and 15% of cases, respectively [11]. Although less frequent, peri-annular complications, such as intracardiac abscess, pseudoaneurysm, or fistula were seen in 3.9% of cases [16]. Different prosthesis designs may also differ in vegetation location. A study evaluating stroke in TAVR-IE observed that 34% of vegetations adhered to the stent frame in SEVs compared to only 19% in BEVs [31]. 

Unfortunately, no specific diagnostic criteria for TAVR-IE have been validated and clinicians typically rely on the modified Duke criteria, even though they are less sensitive in prosthetic valve IE [32]. The Valve Academic Research Consortium-2 in 2012 defined TAVR-IE as the presence of at least one of the following: (i) fulfillment of the modified Duke criteria or (ii) evidence of abscess, paravalvular leak, pus, or vegetation confirmed as secondary to infection by histological or bacteriological studies during a reoperation (iii) or abscess, pus, or vegetation involving a repaired valve on autopsy [33]. Unfortunately, the modified Duke criteria are suboptimal for diagnosing TAVR-IE because TAVR patients are more likely to have indeterminate findings on transthoracic echocardiography (TTE) due to acoustic shadowing and reverberations from the metallic prosthetic valve annulus along with compacted valve calcifications limiting visualization of small vegetations, especially with smaller struts. Of note, the non-linear pathway of perivalvular leaks can lead to underestimation of the significance of a regurgitant lesion [34]. Even more importantly, TTE alone is rarely helpful in differentiating TAVR failure with bystander septicemia of a different origin from true destructive TAVR-IE leading to prosthetic valve failure. Therefore, transesophageal echocardiography (TEE) should be considered in cases of nondiagnostic TTE, high-risk features on TTE, persistent unexplained fever, or concomitant intracardiac devices. When combined with TEE, the sensitivity of echocardiography for TAVR-IE was 67.8% compared to 89.9% for native valve IE [11,35]. Importantly, a comparison between post-TAVR intraprocedural TEE and TEE at the time of TAVR-IE evaluation could help rule out benign lesions, such as peri-annular edema or hematoma [36]. A recent nationwide study by Stortecky et al. reports very poor specificity too, observing that half of all TTE and TEE was interpreted as normal or inconclusive in confirmed TAVR-IE cases [17]. The main challenge with TEE is its limited ability to accurately distinguish small vegetations from thrombi or fibrinous strands.

Given all these echocardiography limitations, 18F-fluorodeoxyglucose positron emission tomography (18F-FDG-PET), multi-detector computed tomography (MDCT), and leukocyte scintigraphy have emerged as promising supplementary imaging modalities in the diagnostic evaluation of TAVR-IE. These additional imaging modalities come with more precise visualization of the aortic valve, annulus and aortic root. In 2015, the European Society of Cardiology (ESC) provided evidence-based guidelines on IE and incorporated the use of 18F-FDG-PET and MDCT into the diagnostic algorithm for clinically suspected IE with indeterminate echocardiographic findings [37]. With regard to TAVR-IE, the addition of 18F-FDG-PET and MDCT may help reclassify as many as 33% of patients with suspected TAVR-IE given their high specificity for TAVR-IE [38]. The limited availability of 18F-FDG-PET and MDCT among non-referral tertiary care centers should further emphasize the importance of prompt referral.

Even though prior literature showed increased physiologic 18F-FDG uptake around the annular ring in the initial months following non-infected SAVR [38], dedicated TAVR studies have not replicated this finding [39]. Potentially, this discrepancy is a result of minimal manipulation of the annulus ring during the positioning of TAVR. Instead, SAVR is surgically adhered to the annulus, which contributes to sterile chronic inflammation. Therefore, the presence of uptake around the prosthetic ring in 18F-FDG-PET after three months post-TAVR should raise suspicion for TAVR-IE, while the uptake pattern (i.e., patchy in TAVR-IE vs. uniform in sterile chronic inflammation) should guide clinicians in the interpretation of 18F-FDG uptake during the initial months. Another relevant advantage of 18F-FDG-PET would be the detection of unexpected extracardiac septic emboli as well as cryptogenic infectious sources [40]. Additionally, early research has demonstrated that bacteria-specific PET radiotracers, such as 18F-fluro-maltohexose, may be useful in distinguishing infection from sterile inflammation, but further research is needed on this modality [41]. MDCT is also often used prior to TAVR to evaluate patient anatomy and offers better visualization of coronary vessels compared to TTE as well as paravalvular complications, such as abscess [42,43]. Despite its constraints inherent to iodinated contrast-associated nephropathy and radiation exposure, MDCT maintains a lower radiation dose compared to standard CT platforms and, importantly, the stochastic risk of radiation exposure is lower in older patients [44] Limited data are available pertaining to TAVR-IE on the role of leukocyte scintigraphy, which detects radiolabeled granulocytes anchored on the valve, yielding a high specificity for prosthetic valve IE [45,46]. While this would be a very attractive option, especially in cases of ambiguous 18F-FDG uptake patterns, the main current limitation of leukocyte scintigraphy lies in its low availability among non-referral centers. Finally, cardiac MRI has not been well studied in TAVR-IE, but valve-related artifacts likely limit its utility.

## 6. Management and Outcomes

TAVR-IE has been associated with increased rates of serious complications including acute heart failure, severe valve dysfunction, renal dysfunction, severe sepsis and septic shock, and coronary as well as systemic embolization [11,12]. The rates of in-hospital mortality are high in this population ranging from 20–64% [16,47,48,49]. Accordingly, it is paramount for these patients to be managed at tertiary care hospitals equipped to deal with these complications. 

**Figure 2:**
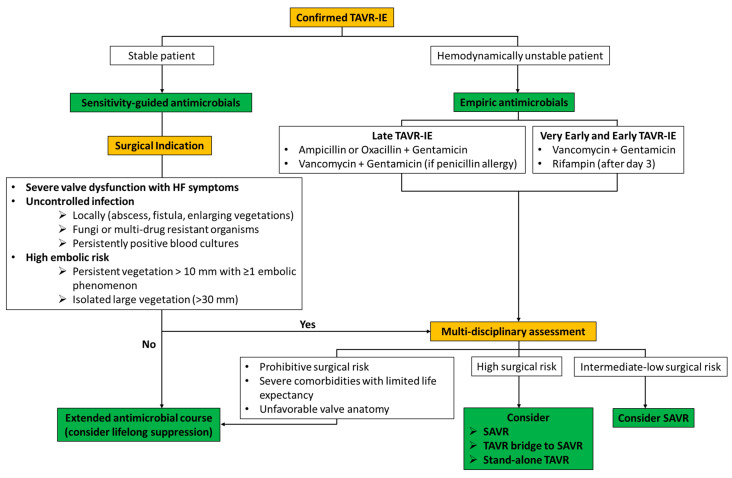
Proposed management algorithm of TAVR-related IE (TAVR-IE). Late TAVR-IE, beyond 1-year post-TAVR; early TAVR-IE, within 1-year post-TAVR; SAVR, surgical aortic valve replacement.

The appropriate management of these patients remains debatable and is multifaceted necessitating a collaborative multidisciplinary heart valve team approach across the departments of cardiology, structural intervention, infectious disease and cardiothoracic surgery. There is a dearth of data comparing conservative targeted antibiotic strategy alone with surgical management; hence, the optimal management strategy needs to be formulated on a case-by-case basis. A therapeutic algorithm is shown in Figure 2.

Early diagnosis with a high index of suspicion and timely initiation of antibiotic therapy is key to preventing devastating complications. Expedite identification of the culprit agent remains the cornerstone of TAVR-IE management, but standard culture-based methods have limited sensitivity particularly after improper antibiotic initiation or in case of fastidious organisms (including common small colony variants of *Staphylococcus aureus*, which can act as a facultative intracellular pathogen) [50]. Notably, blood cultures are imperfect indicators of the actual causative agents, as demonstrated by a systematic review by Oberbach et al. who showed that 1/3 of patients had the same pathogens in blood and prosthetic valves [51]. Given its importance, pathogen detection should be aided with molecular-based detection methods, which have the added benefit of providing the virulence profile of the culprit pathogen [51]. In fact, the highly prevalent Gram-positive cocci (GPC) possess a remarkable ability to acquire resistance genes and targeted antibiotic regimens for widely emerging drug-resistant GPC have not been well studied [52]. Therefore, antibiotic-independent approaches (e.g., targeted control of quorum sensing-specific molecules) to reduce biofilm formation, prevent the transfer of resistance genes, reduce the exponential microbial growth and unmask pathogens have been studied, but require further validation [51]. Upfront empiric antibiotics in hemodynamically unstable patients usually comprise a combination of aminoglycoside and vancomycin [37]. The long-term antibiotic treatment should be guided by microbiological profiles available from blood cultures and hospital-wide antimicrobial sensitivity testing data. Implanted prosthetic material acts as a nidus for bacterial colonization, making eradication significantly challenging even with targeted antibiotic therapy. An antibiotic regimen is administered for a longer duration of at least 6 weeks which is extrapolated from the management of prosthetic valve endocarditis, as appropriate duration is not well established for TAVR-IE. Some evidence suggests that residual vegetation after a 6-week course of tailored antibiotics in the absence of clinically or microbiologically persistent TAVR-IE is not to be considered treatment failure [53]. However, repeat follow-up imaging would be advised to confirm the presence of vegetation stable in size.

These patients also pose a high risk for hospital-associated infections, prolonged hospitalization and physical deconditioning which should be prevented by aggressive physical therapy, antibiotic stewardship and appropriate hand hygiene and room disinfection per individualized hospital policies. 

Definitive therapy would ideally include prosthetic valve replacement, but these patients are often too critically ill or unfit to undergo surgery. Lifelong suppressive antibiotics are commonly employed, but progressive deterioration of the prosthetic valve is to be expected as well as the selection for antibiotic-resistant organisms. Extrapolating from prosthetic valve IE guidelines, surgery could be offered in patients with resistant infections (e.g., abscess, enlarging vegetation, fistula), cardiogenic shock due to acute valvular pathology, high embolic risk (e.g., large vegetations), persistent bacteremia despite appropriate antibiotics, and fungal organisms. Satellite lesions to mitral or tricuspid valves and coronary or systemic embolization are also indications for surgical management. Despite these indications, low rates of surgical intervention (<20% on average) are consistently reported across studies with relatively small sample sizes [10,18,22,24]. Although the generalizability of these results is limited, the low surgical rates are often attributed to prohibitive surgical risks, increased complexity of surgical interventions and decreased availability of these procedures across all hospitals. It is also important to note that cardiac surgery is not associated with improvement in in-hospital or all-cause mortality at 1 year compared with conservative management alone [18,48]. The increased rates of mortality should be clearly stated to patients and family members, whereas early introduction of palliative care is encouraged in patients not candidates for surgical management.

Long-term follow-up data are limited but all-cause mortality at 1 year is increased and mortality rates are higher than those reported for native and surgical valve IE [54]. Therefore, TAVR-IE should be considered a distinct entity when compared with prosthetic valve endocarditis. An overview of the reported mortality in clinical trials and large registry analyses is shown in Table 3. 

A systematic review of historical TAVR data revealed an abysmal in-hospital mortality of 34% in a very high-risk profile of TAVR patients, while pooled data of PARTNER trials found TAVR-IE to be an independent risk factor for mortality at 5 years [9,22]. A multicenter, international TAVR registry of 250 TAVR-IE patients reported poor long-term prognosis with mortality in about two-thirds of patients at 5-year follow up [55]. However, temporal trends have shown an encouraging progressive decline in rates of in-hospital and 1-year mortality over time. Given lower sample sizes and the retrospective nature of most studies, no specific recommendations or guidelines have been established for the management of these patients. Hence, the appropriate treatment strategy remains ambiguous, calling for large prospective registries to define the role and indications of surgery in these patients and put forward an optimal treatment algorithm.

## 7. Prevention

Prevention of TAVR-IE remains the cornerstone of TAVR patients’ management, in light of the lack of evidence-based life-saving therapies and overall high mortality. Both the American Heart Association (AHA) and the Centers for Disease Control and Prevention recommend routine use of a one-time dose of pre-procedural antimicrobial prophylaxis (Class I), with the exception of situations where it was inadvertently missed, in which case the dose can be administered within 2 h after the procedure [20,56]. The AHA did not provide any strong recommendations for specific perioperative antibiotics, which should be tailored according to local resistance patterns. In contrast, the ESC advises periprocedural antimicrobial prophylaxis with IV first-generation cephalosporin from 1 h prior to the procedure until 48 h after TAVR (Class IIa) [37]. Longer prophylactic regimens have shown no advantage and were associated with a higher risk of *C. difficile* infection [57]. Remarkable variation in dosage and frequency has been reported by different centers [54], demonstrating a lack of universal agreement in this field. The latest AHA guidelines, which recommend the use of cephalosporin before TAVR, downgraded their level of evidence from B (moderate quality) to C-LD (limited data) with regard to IE prophylaxis in TAVR [20]. These guidelines used data largely extrapolated from the SAVR literature. More recent guidelines from the International Society for Cardiovascular Infectious Diseases (ISCID) advocate for adapted prophylaxis with activity against the highly prevalent enterococci in TAVR-IE [58]. In fact, cephalosporins traditionally used for pre-TAVR prophylaxis lack meaningful activity against *Enterococci*, which could potentially explain the AHA downgrading of its recommendation. In a large registry analysis including 7203 patients, 50% of periprocedural TAVR-IE was caused by organisms not susceptible to standard periprocedural antibiotic regimens [17]. Therefore, ISCID recommends an enterococcal-active agent, such as amoxicillin-clavulanic acid (or ampicillin-sulbactam) in penicillin-nonallergic patients and vancomycin in penicillin-allergic patients, within 60 min before the arterial puncture and eventually followed by a second dose in case of procedures longer than 2 h [58]. Given the low likelihood of future dedicated randomized clinical trials, further observational studies are needed to determine optimal regimens (e.g., single vs. double dosing). 

Even though TAVR performed in a catheterization laboratory was not associated with more TAVR-IE compared to an operating room, [11] strict infection prevention measures should be enacted even in the catheterization laboratory to minimize risk. Regardless, since ~50% of TAVR-IE cases are likely healthcare-associated, [11], some investigators have stressed the need to limit unnecessary healthcare-associated procedures that could promote bacteremia. Importantly, active infections should warrant deferral of elective TAVR procedures. The 2015 ESC Guidelines also recommend preoperative screening of nasal *Staphylococcus aureus* (class I) and decolonization with both whole-body chlorhexidine showering and nasal mupirocin for 5 days similarly to SAVR [37]. These strategies should be enforced in known *S. aureus* carriers or patients with body mass index > 30 kg/m^2^ and concomitant diabetes.

Lastly, post-TAVR antibiotic prophylaxis should be utilized in all patients before dental procedures involving manipulation of the oral mucosa or gingiva. While the incidence of TAVR-IE after other invasive procedures (respiratory, gastrointestinal, urogenital tract) is still unknown, extensive use of antibiotic prophylaxis is no longer recommended due to emerging antibiotic resistance, potential adverse drug reactions and highly inefficient number needed to treat [58].

## 8. Future Perspectives

Improving device technology, reducing the invasiveness of procedures, increasing operators’ skills, and streamlining periprocedural care may hopefully lead to lower numbers of early TAVR-IE. A possible trade-off of greater expected life expectancy of future low-surgical-risk TAVR patients may be an increase in late TAVR-IE. Given its overall low incidence, the best available recommendations on TAVR-IE have been generated from secondary endpoints of randomized clinical trials. However, dedicated randomized trials to study TAVR-IE management (both medical and surgical) should become a primary focus of the scientific community. As an important adjunct, the development of collaborative, multi-center prospective real-world studies will be of paramount importance to facilitate the production of generalizable results and shed light on the numerous complexities pertaining TAVR-IE, including diagnostic risk tools, refined diagnostic criteria, more effective periprocedural antibiotic prophylaxis (including the testing of new TAVR device iterations with antibacterial biomaterials), more sophisticated imaging modalities (such as leukocyte scintigraphy or the use of bacteria-specific PET radiotracers), and standardized surgical approaches. All these combined efforts should eventually enable the scientific community to create specific TAVR-IE guidelines out of high-quality TAVR-IE data as opposed to extrapolating from the SAVR literature.

Importantly, the scientific community should avoid a “one-size-fits-all” approach and, instead, focus its efforts on taking a deep dive into the different TAVR patient subpopulations in an effort to promote individualized care for TAVR-IE. In fact, TAVR-IE occurring in a young patient with bicuspid aortic valve is likely to require completely different care compared to an old patient with metastatic carcinoma. However, more evidence is needed to provide clinicians with the tools to confidently take care of this complex patient population.

## 9. Gaps in Evidence

Despite accruing experience with TAVR, evidence for optimal diagnostic pathways, effective prevention, and appropriate management of TAVR-IE is largely extrapolated from non-TAVR populations and multiple gaps in evidence remain. From a diagnostic standpoint, TAVR-IE continues to be a challenging diagnosis even with TEE and studies investigating the potential beneficial role of early multimodality imaging (18F-FDG-PET and/or MDCT) would be helpful. From a preventive standpoint, dedicated studies on TAVR patients may shed additional light on the optimal dosing and frequency of antibiotic prophylaxis given the current lack of universal agreement. From a therapeutic standpoint, considering the relatively low incidence of TAVR-IE and the difficult enrollment of a tightly selected cohort, pragmatic trials may help develop the optimal therapeutic strategy, particularly for non-palliative TAVR-IE patients. Both the ideal strategy (ViV-TAVR stand-alone vs. ViV-TAVR bridge to SAVR vs. SAVR) and timing are likely to differ between different patient phenotypes, but additional evidence is needed to further guide decision-making.

## 10. Conclusions

TAVR-IE is a relatively rare event after TAVR. However, the exponentially increasing patient population amenable to TAVR will lead to more patients at risk of developing this life-threatening complication and adequate prophylaxis should be strictly enforced. While challenging, early recognition with multimodality imaging is key to promptly initiating appropriate management. TAVR-IE remains extremely controversial from a therapeutic standpoint, given the highly heterogeneous recommendations. Sensitivity-guided antimicrobials and multi-disciplinary procedural management are of paramount importance (Graphical Abstract). The majority of TAVR-IE patients with surgical indications are unlikely to be offered SAVR because of either acute hemodynamic instability or high surgical risk (driven by advanced age and complex comorbidities). The caring team should evaluate the feasibility of rescue ViV-TAVR (stand-alone or bridge to SAVR) early on, before the potential development of organ failure.

## Figures and Tables

**Table 1:** Incidence of very early (within 30 days post-TAVR), early (between 31 and 365 days post-TAVR) and late (beyond 1-year post-TAVR) TAVR-related infective endocarditis (TAVR-IE).

	Incidence of TAVR-IE		
Trial	Very Early	Early	Late
*PARTNER 3*			
TAVR	0.0%	0.2%	0.2%
SAVR	0.2%	0.5%	0.9%
*PARTNER 2*			
TAVR	0.0%	0.8%	1.2%
SAVR	0.0%	0.7%	0.7%
*PARTNER 1*			
TAVR	0.0%	1.0%	1.5%
SAVR	0.3%	1.1%	1.0%
*Evolut Low-Risk Trial*			
TAVR	0.1%	0.2%	-
SAVR	0.2%	0.4%	-

TAVR, transcatheter aortic valve replacement; SAVR, surgical aortic valve replacement; TAVR-IE, TAVR-related infective endocarditis.

**Table 2:** Timeline and risk factors of causative organisms of TAVR-related infective endocarditis (TAVR-IE).

Causative Organism	Incidence	Time from TAVR ^a^	Risk Factors	Strength of Association ^b^	Level of Evidence ^c^
**Most Common**					
*Staphylococcus*	22–30%	>1-year post-TAVR	Soft tissue infection, vascular access	High	B–NR
*Streptococcus*	20–30%	>1-year post-TAVR	Dental procedures, low-risk TAVR	Low	B–NR
*Enterococcus*	15–25%	<100 days post-TAVR	Groin colonization ^1^, frequent healthcare use, higher antibiotic exposure	Moderate	B–NR
**Least common**					
Gram negative	<5%	Median time—1.1 months	Nosocomial infections	N/A	C–EO
Fungal	0.8–3%	Limited data	Drug abuse	N/A	C–LD

^a^. Time from TAVR: outlines the more prevalent organism for a particular time period. ^b^. Strength of Association: defined as reported incidence in TARV-IE patients. High—over 10%, Moderate—incidence 5% to 10%, Low—incidence less than 5%, N/A insufficient data. ^c^. Levels of evidence as outlined in the 2022 AHA/ACC/HFSA Guideline for the management of Heart Failure. Level A: high quality. Level B–R: moderate quality from randomized control trials. Level B–NR: moderate quality from well-designed, nonrandomized studies. Level C–LD: studies with limitations of design or execution. Level C–EO: expert opinion. ^1^ Defined as the detection of Enterococcus on skin swab prior to prophylactic antibiotics and groin sterilization before transfemoral intervention.

**Table 3:** Reported mortality data after TAVR-related infective endocarditis (TAVR-IE).

TAVR-IE Outcomes					
Study	Surgical Aortic Valve Explant	Mortality			
		Very Early	Early	Late	Very Late
Mentias et al., 2020 [10]	3.8%	18.5%	45.6%	-	-
Stortecky et al., 2020 [17]	-	26.0%	43.7%	-	-
Fauchier et al., 2020 [13]	-	18.7%	32.8%	-	-
del Val et al., 2020 [55]	-	-	-	-	62.5%
Regueiro et al., 2019 [21]	10.8%	-	52.0%	67.0%	-

Definitions: Very early: under 30 days post-TAVR. Early: between 31 and 365 days post-TAVR. Late: 1–2 years post-TAVR. Very Late: 2–5 years post-TAVR.

## Data Availability

No new data were created or analyzed in this study. Data sharing is not applicable to this article.

## References

[1] Coffey S., Cairns B.J., Iung B. (2016). The modern epidemiology of heart valve disease. Heart.

[2] Makkar R.R., Fontana G.P., Jilaihawi H., Kapadia S., Pichard A.D., Douglas P.S., Thourani V.H., Babaliaros V.C., Webb J.G., Herrmann H.C. (2012). Transcatheter aortic-valve replacement for inoperable severe aortic stenosis. N. Engl. J. Med..

[3] Leon M.B., Smith C.R., Mack M.J., Makkar R.R., Svensson L.G., Kodali S.K., Thourani V.H., Tuzcu E.M., Miller D.C., Herrmann H.C. (2016). Transcatheter or Surgical Aortic-Valve Replacement in Intermediate-Risk Patients. N. Engl. J. Med..

[4] Reardon M.J., Van Mieghem N.M., Popma J.J., Kleiman N.S., Sondergaard L., Mumtaz M., Adams D.H., Deeb G.M., Maini B., Gada H. (2017). Surgical or Transcatheter Aortic-Valve Replacement in Intermediate-Risk Patients. N. Engl. J. Med..

[5] Mack M.J., Leon M.B., Thourani V.H., Makkar R., Kodali S.K., Russo M., Kapadia S.R., Malaisrie S.C., Cohen D.J., Pibarot P. (2019). Transcatheter Aortic-Valve Replacement with a Balloon-Expandable Valve in Low-Risk Patients. N. Engl. J. Med..

[6] Popma J.J., Deeb G.M., Yakubov S.J., Mumtaz M., Gada H., O’Hair D., Bajwa T., Heiser J.C., Merhi W., Kleiman N.S. (2019). Transcatheter Aortic-Valve Replacement with a Self-Expanding Valve in Low-Risk Patients. N. Engl. J. Med..

[7] Mentias A., Saad M., Desai M.Y., Krishnaswamy A., Menon V., Horwitz P.A., Kapadia S., Sarrazin M.V. (2021). Transcatheter Versus Surgical Aortic Valve Replacement in Patients with Rheumatic Aortic Stenosis. J. Am. Coll. Cardiol..

[8] Mentias A., Saad M., Menon V., Reed G.W., Popovic Z., Johnston D., Rodriguez L., Gillinov M., Griffin B., Jneid H. (2023). Transcatheter vs Surgical Aortic Valve Replacement in Pure Native Aortic Regurgitation. Ann. Thorac. Surg..

[9] Summers M.R., Leon M.B., Smith C.R., Kodali S.K., Thourani V.H., Herrmann H.C., Makkar R.R., Pibarot P., Webb J.G., Leipsic J. (2019). Prosthetic Valve Endocarditis After TAVR and SAVR: Insights from the PARTNER Trials. Circulation.

[10] Mentias A., Girotra S., Desai M.Y., Horwitz P.A., Rossen J.D., Saad M., Panaich S., Kapadia S., Sarrazin M.V. (2020). Incidence, Predictors, and Outcomes of Endocarditis After Transcatheter Aortic Valve Replacement in the United States. JACC Cardiovasc. Interv..

[11] Regueiro A., Linke A., Latib A., Ihlemann N., Urena M., Walther T., Husser O., Herrmann H.C., Nombela-Franco L., Cheema A.N. (2016). Association Between Transcatheter Aortic Valve Replacement and Subsequent Infective Endocarditis and In-Hospital Death. JAMA.

[12] Kolte D., Goldsweig A., Kennedy K.F., Abbott J.D., Gordon P.C., Sellke F.W., Ehsan A., Sodha N., Sharaf B.L., Aronow H.D. (2018). Comparison of Incidence, Predictors, and Outcomes of Early Infective Endocarditis after Transcatheter Aortic Valve Implantation Versus Surgical Aortic Valve Replacement in the United States. Am. J. Cardiol..

[13] Fauchier L., Bisson A., Herbert J., Lacour T., Bourguignon T., Etienne C.S., Bernard A., Deharo P., Bernard L., Babuty D. (2020). Incidence and outcomes of infective endocarditis after transcatheter aortic valve implantation versus surgical aortic valve replacement. Clin. Microbiol. Infect..

[14] Medranda G.A., Rogers T., Ali S.W., Zhang C., Shea C., Sciandra K.A., Case B.C., Forrestal B.J., Sutton J.A., McFadden E.P. (2022). Prosthetic valve endocarditis after transcatheter aortic valve replacement in low-risk patients. Catheter. Cardiovasc. Interv..

[15] Durko A.P., Osnabrugge R.L., Van Mieghem N.M., Milojevic M., Mylotte D., Nkomo V.T., Pieter Kappetein A. (2018). Annual number of candidates for transcatheter aortic valve implantation per country: Current estimates and future projections. Eur. Heart J..

[16] Del Val D., Abdel-Wahab M., Linke A., Durand E., Ihlemann N., Urena M., Pellegrini C., Giannini F., Landt M., Auffret V. (2021). Temporal Trends, Characteristics, and Outcomes of Infective Endocarditis After Transcatheter Aortic Valve Replacement. Clin. Infect. Dis..

[17] Stortecky S., Heg D., Tueller D., Pilgrim T., Muller O., Noble S., Jeger R., Toggweiler S., Ferrari E., Taramasso M. (2020). Infective Endocarditis After Transcatheter Aortic Valve Replacement. J. Am. Coll. Cardiol..

[18] Mangner N., Woitek F., Haussig S., Schlotter F., Stachel G., Hollriegel R., Wilde J., Lindner A., Holzhey D., Leontyev S. (2016). Incidence, Predictors, and Outcome of Patients Developing Infective Endocarditis Following Transfemoral Transcatheter Aortic Valve Replacement. J. Am. Coll. Cardiol..

[19] Murdoch D.R., Corey G.R., Hoen B., Miro J.M., Fowler V.G., Jr Bayer A.S., Karchmer A.W., Olaison L., Pappas P.A., Moreillon P. (2009). Clinical presentation, etiology, and outcome of infective endocarditis in the 21st century: The International Collaboration on Endocarditis-Prospective Cohort Study. Arch. Intern. Med..

[20] Alexis S.L., Malik A.H., George I., Hahn R.T., Khalique O.K., Seetharam K., Bhatt D.L., Tang G.H.L. (2020). Infective Endocarditis After Surgical and Transcatheter Aortic Valve Replacement: A State of the Art Review. J. Am. Heart Assoc..

[21] Regueiro A., Linke A., Latib A., Rodés-Cabau J., Anguita M., de la Figuera M., Cabeza A.I.P., Fernández C.S., Munoz-Garcia E., Munoz-Garcia M. (2019). Infective Endocarditis Following Transcatheter Aortic Valve Replacement: Comparison of Balloon- Versus Self-Expandable Valves. Circ. Cardiovasc. Interv..

[22] Amat-Santos I.J., Messika-Zeitoun D., Eltchaninoff H., Kapadia S., Lerakis S., Cheema A.N., Gutierrez-Ibanes E., Munoz-Garcia A.J., Pan M., Webb J.G. (2015). Infective endocarditis after transcatheter aortic valve implantation: Results from a large multicenter registry. Circulation.

[23] Prasitlumkum N., Vutthikraivit W., Thangjui S., Leesutipornchai T., Kewcharoen J., Riangwiwat T., Dworkin J. (2020). Epidemiology of infective endocarditis in transcatheter aortic valve replacement: Systemic review and meta-analysis. J. Cardiovasc. Med..

[24] Bjursten H., Rasmussen M., Nozohoor S., Gotberg M., Olaison L., Ruck A., Ragnarsson S. (2019). Infective endocarditis after transcatheter aortic valve implantation: A nationwide study. Eur. Heart J..

[25] Zakhour J., Allaw F., Kalash S., Wehbe S., Kanj S.S. (2023). Infective Endocarditis after Transcatheter Aortic Valve Replacement: Challenges in the Diagnosis and Management. Pathogens.

[26] Tinica G., Tarus A., Enache M., Artene B., Rotaru I., Bacusca A., Burlacu A. (2020). Infective endocarditis after TAVI: A meta-analysis and systematic review of epidemiology, risk factors and clinical consequences. Rev. Cardiovasc. Med..

[27] Cahill T.J., Baddour L.M., Habib G., Hoen B., Salaun E., Pettersson G.B., Schafers H.J., Prendergast B.D. (2017). Challenges in Infective Endocarditis. J. Am. Coll. Cardiol..

[28] Del Val D., Abdel-Wahab M., Mangner N., Durand E., Ihlemann N., Urena M., Pellegrini C., Giannini F., Gasior T., Wojakowski W. (2022). Infective Endocarditis Caused by Staphylococcus aureus After Transcatheter Aortic Valve Replacement. Can. J. Cardiol..

[29] Panagides V., Abdel-Wahab M., Mangner N., Durand E., Ihlemann N., Urena M., Pellegrini C., Giannini F., Scislo P., Huczek Z. (2022). Very early infective endocarditis after transcatheter aortic valve replacement. Clin. Res. Cardiol..

[30] Salaun E., Sportouch L., Barral P.A., Hubert S., Lavoute C., Casalta A.C., Pradier J., Ouk D., Casalta J.P., Lambert M. (2018). Diagnosis of Infective Endocarditis After TAVR: Value of a Multimodality Imaging Approach. JACC Cardiovasc. Imaging.

[31] Del Val D., Abdel-Wahab M., Mangner N., Durand E., Ihlemann N., Urena M., Pellegrini C., Giannini F., Gasior T., Wojakowski W. (2021). Stroke Complicating Infective Endocarditis After Transcatheter Aortic Valve Replacement. J. Am. Coll. Cardiol..

[32] Perez-Vazquez A., Farinas M.C., Garcia-Palomo J.D., Bernal J.M., Revuelta J.M., Gonzalez-Macias J. (2000). Evaluation of the Duke criteria in 93 episodes of prosthetic valve endocarditis: Could sensitivity be improved?. Arch Intern Med.

[33] Kappetein A.P., Head S.J., Genereux P., Piazza N., van Mieghem N.M., Blackstone E.H., Brott T.G., Cohen D.J., Cutlip D.E., van Es G.A. (2012). Updated standardized endpoint definitions for transcatheter aortic valve implantation: The Valve Academic Research Consortium-2 consensus document. Eur. Heart J..

[34] Kinno M., Cantey E.P., Rigolin V.H. (2019). The transition from transesophageal to transthoracic echocardiography during transcatheter aortic valve replacement: An evolving field. J. Echocardiogr..

[35] Wang A., Athan E., Pappas P.A., Fowler V.G., Jr Olaison L., Pare C., Almirante B., Munoz P., Rizzi M., Naber C. (2007). Contemporary clinical profile and outcome of prosthetic valve endocarditis. JAMA.

[36] Dilsizian V., Budde R.P.J., Chen W., Mankad S.V., Lindner J.R., Nieman K. (2022). Best Practices for Imaging Cardiac Device-Related Infections and Endocarditis: A JACC: Cardiovascular Imaging Expert Panel Statement. JACC Cardiovasc. Imaging.

[37] Habib G., Lancellotti P., Antunes M.J., Bongiorni M.G., Casalta J.P., Del Zotti F., Dulgheru R., El Khoury G., Erba P.A., Iung B. (2015). 2015 ESC Guidelines for the management of infective endocarditis: The Task Force for the Management of Infective Endocarditis of the European Society of Cardiology (ESC). Endorsed by: European Association for Cardio-Thoracic Surgery (EACTS), the European Association of Nuclear Medicine (EANM). Eur. Heart J..

[38] Wahadat A.R., Tanis W., Swart L.E., Scholtens A., Krestin G.P., van Mieghem N., Schurink C.A.M., van der Spoel T.I.G., van den Brink F.S., Vossenberg T. (2021). Added value of (18)F-FDG-PET/CT and cardiac CTA in suspected transcatheter aortic valve endocarditis. J. Nucl. Cardiol..

[39] Del Val D., Trottier M., Alperi A., Muntane-Carol G., Faroux L., Delarochelliere R., Paradis J.M., Dumont E., Kalavrouziotis D., Mohammadi S. (2020). (18)F-Fluorodeoxyglucose Uptake Pattern in Noninfected Transcatheter Aortic Valves. Circ. Cardiovasc. Imaging.

[40] Chen W., Sajadi M.M., Dilsizian V. (2018). Merits of FDG PET/CT and Functional Molecular Imaging Over Anatomic Imaging with Echocardiography and CT Angiography for the Diagnosis of Cardiac Device Infections. JACC Cardiovasc. Imaging.

[41] Takemiya K., Ning X., Seo W., Wang X., Mohammad R., Joseph G., Titterington J.S., Kraft C.S., Nye J.A., Murthy N. (2019). Novel PET and Near Infrared Imaging Probes for the Specific Detection of Bacterial Infections Associated With Cardiac Devices. JACC Cardiovasc. Imaging.

[42] De Cecco C.N., Bastarrika G., Arraiza M., Maurizi Enrici M., Pueyo J., Muscogiuri G., Fina P., Anselmi A., Di Girolamo M., David V. (2012). Dual source CT: State of the art in the depiction of coronary arteries anatomy, anatomical variants and myocardial segments. Minerva Cardioangiol..

[43] Hryniewiecki T., Zatorska K., Abramczuk E., Zakrzewski D., Szymanski P., Kusmierczyk M., Michalowska I. (2019). The usefulness of cardiac CT in the diagnosis of perivalvular complications in patients with infective endocarditis. Eur. Radiol..

[44] Horgan S.J., Mediratta A., Gillam L.D. (2020). Cardiovascular Imaging in Infective Endocarditis: A Multimodality Approach. Circ. Cardiovasc. Imaging.

[45] Gomes A., Glaudemans A., Touw D.J., van Melle J.P., Willems T.P., Maass A.H., Natour E., Prakken N.H.J., Borra R.J.H., van Geel P.P. (2017). Diagnostic value of imaging in infective endocarditis: A systematic review. Lancet Infect. Dis..

[46] Hyafil F., Rouzet F., Lepage L., Benali K., Raffoul R., Duval X., Hvass U., Iung B., Nataf P., Lebtahi R. (2013). Role of radiolabelled leucocyte scintigraphy in patients with a suspicion of prosthetic valve endocarditis and inconclusive echocardiography. Eur. Heart J. Cardiovasc. Imaging.

[47] Moriyama N., Laakso T., Biancari F., Raivio P., Jalava M.P., Jaakkola J., Dahlbacka S., Kinnunen E.M., Juvonen T., Husso A. (2019). Prosthetic valve endocarditis after transcatheter or surgical aortic valve replacement with a bioprosthesis: Results from the FinnValve Registry. EuroIntervention.

[48] Mangner N., Leontyev S., Woitek F.J., Kiefer P., Haussig S., Binner C., Mende M., Schlotter F., Stachel G., Hollriegel R. (2018). Cardiac Surgery Compared with Antibiotics Only in Patients Developing Infective Endocarditis After Transcatheter Aortic Valve Replacement. J. Am. Heart Assoc..

[49] Latib A., Naim C., De Bonis M., Sinning J.M., Maisano F., Barbanti M., Parolari A., Lorusso R., Testa L., Actis Dato G.M. (2014). TAVR-associated prosthetic valve infective endocarditis: Results of a large, multicenter registry. J. Am. Coll. Cardiol..

[50] Oberbach A., Schlichting N., Feder S., Lehmann S., Kullnick Y., Buschmann T., Blumert C., Horn F., Neuhaus J., Neujahr R. (2017). New insights into valve-related intramural and intracellular bacterial diversity in infective endocarditis. PLoS ONE.

[51] Oberbach A., Schlichting N., Hagl C., Lehmann S., Kullnick Y., Friedrich M., Kohl U., Horn F., Kumbhari V., Loffler B. (2023). Four decades of experience of prosthetic valve endocarditis reflect a high variety of diverse pathogens. Cardiovasc. Res..

[52] Jean S.S., Liu I.M., Hsieh P.C., Kuo D.H., Liu Y.L., Hsueh P.R. (2023). Off-label use versus formal recommendations of conventional and novel antibiotics for the treatment of infections caused by multidrug-resistant bacteria. Int. J. Antimicrob. Agents.

[53] Houard V., Porte L., Delon C., Carrie D., Delobel P., Galinier M., Lairez O., Lavie-Badie Y. (2020). Prognostic value of residual vegetation after antibiotic treatment for infective endocarditis: A retrospective cohort study. Int. J. Infect. Dis..

[54] Del Val D., Panagides V., Mestres C.A., Miro J.M., Rodes-Cabau J. (2023). Infective Endocarditis After Transcatheter Aortic Valve Replacement: JACC State-of-the-Art Review. J. Am. Coll. Cardiol..

[55] Del Val D., Linke A., Abdel-Wahab M., Latib A., Ihlemann N., Urena M., Won-Keun K., Husser O., Herrmann H.C., Nombela-Franco L. (2020). Long-Term Outcomes After Infective Endocarditis After Transcatheter Aortic Valve Replacement. Circulation.

[56] Berrios-Torres S.I., Umscheid C.A., Bratzler D.W., Leas B., Stone E.C., Kelz R.R., Reinke C.E., Morgan S., Solomkin J.S., Mazuski J.E. (2017). Centers for Disease Control and Prevention Guideline for the Prevention of Surgical Site Infection, 2017. JAMA Surg..

[57] Gomes B., Geis N.A., Leuschner F., Meder B., Konstandin M., Katus H.A., Bekeredjian R. (2018). Periprocedural antibiotic treatment in transvascular aortic valve replacement. J. Interv. Cardiol..

[58] Conen A., Stortecky S., Moreillon P., Hannan M.M., Franzeck F.C., Jeger R., Widmer A.F. (2021). A review of recommendations for infective endocarditis prevention in patients undergoing transcatheter aortic valve implantation. EuroIntervention.

